# Supplementary material for: Saccharomyces boulardii (CNCM I-745) improves intestinal damage in sepsis by remodeling intestinal flora structure
Source: Front Cell Infect Microbiol. 2025 Jul 31;15:1602792. doi: 10.3389/fcimb.2025.1602792 (PMC12350329; doi:10.3389/fcimb.2025.1602792)
Supplement: Supplementary file 1 [file DataSheet1.zip › summary/summary/8_Advanced_analyse/2_PICRUSt2_result/KEGG/Group.DW4_vs_BW4/PICRUSt2_KO_Group.DW4_vs_BW4_diff.pdf]

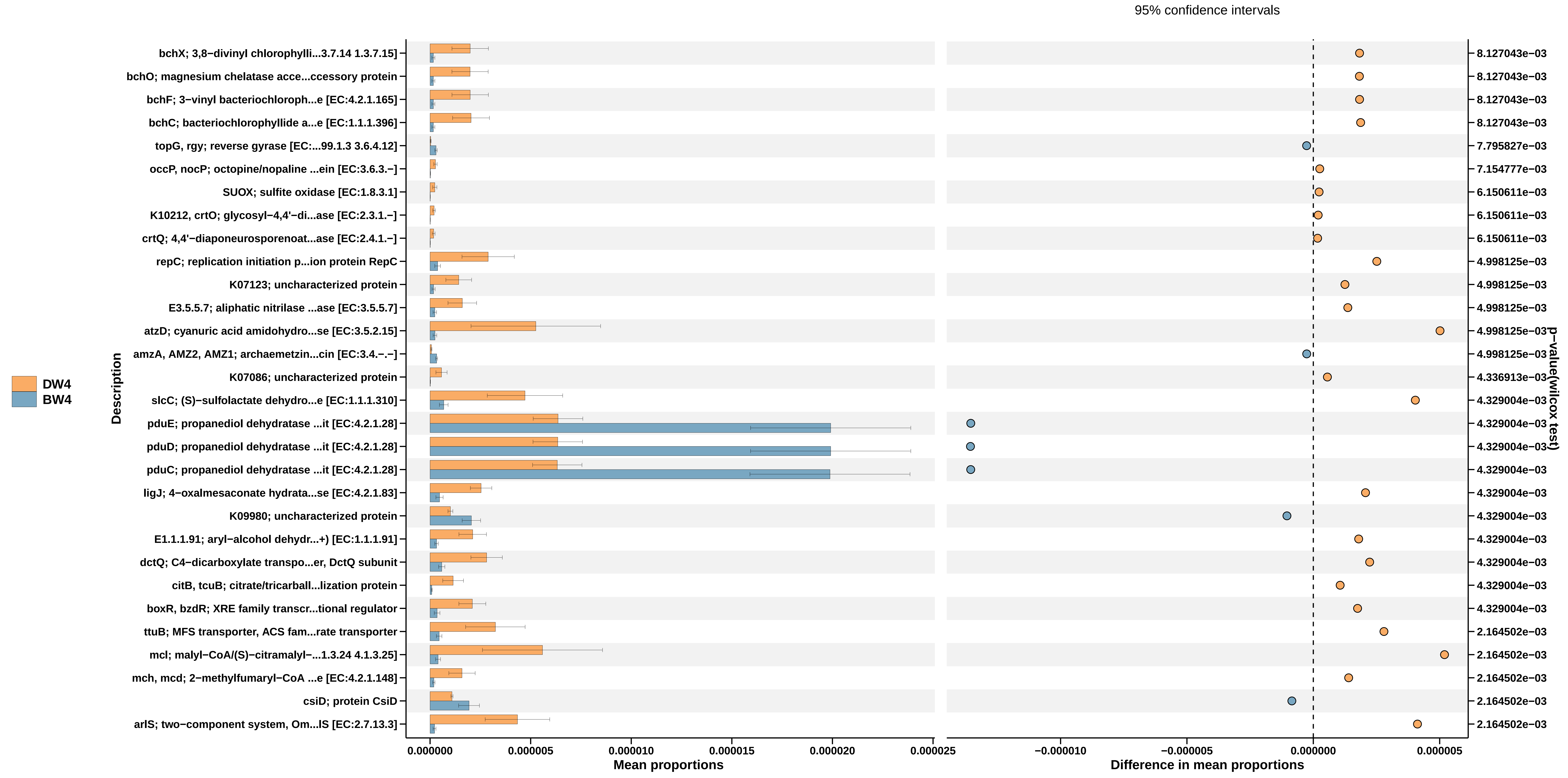

95% confidence intervals
DW4
BW4
Description
bchX; 3,8−divinyl chlorophylli...3.7.14 1.3.7.15]
bchO; magnesium chelatase acce...ccessory protein
bchF; 3−vinyl bacteriochloroph...e [EC:4.2.1.165]
bchC; bacteriochlorophyllide a...e [EC:1.1.1.396]
topG, rgy; reverse gyrase [EC:...99.1.3 3.6.4.12]
occP, nocP; octopine/nopaline ...ein [EC:3.6.3.−]
SUOX; sulfite oxidase [EC:1.8.3.1]
K10212, crtO; glycosyl−4,4'−di...ase [EC:2.3.1.−]
crtQ; 4,4'−diaponeurosporenoat...ase [EC:2.4.1.−]
repC; replication initiation p...ion protein RepC
K07123; uncharacterized protein
E3.5.5.7; aliphatic nitrilase ...ase [EC:3.5.5.7]
atzD; cyanuric acid amidohydro...se [EC:3.5.2.15]
amzA, AMZ2, AMZ1; archaemetzin...cin [EC:3.4.−.−]
K07086; uncharacterized protein
slcC; (S)−sulfolactate dehydro...e [EC:1.1.1.310]
pduE; propanediol dehydratase ...it [EC:4.2.1.28]
pduD; propanediol dehydratase ...it [EC:4.2.1.28]
pduC; propanediol dehydratase ...it [EC:4.2.1.28]
ligJ; 4−oxalmesaconate hydrata...se [EC:4.2.1.83]
K09980; uncharacterized protein
E1.1.1.91; aryl−alcohol dehydr...+) [EC:1.1.1.91]
dctQ; C4−dicarboxylate transpo...er, DctQ subunit
citB, tcuB; citrate/tricarball...lization protein
boxR, bzdR; XRE family transcr...tional regulator
ttuB; MFS transporter, ACS fam...rate transporter
mcl; malyl−CoA/(S)−citramalyl−...1.3.24 4.1.3.25]
mch, mcd; 2−methylfumaryl−CoA ...e [EC:4.2.1.148]
csiD; protein CsiD
arlS; two−component system, Om...lS [EC:2.7.13.3]
0.000000
0.000005
0.000010
0.000015
0.000020
0.000025
Mean proportions
−0.000010
−0.000005
0.000000
0.000005
Difference in mean proportions
p−value(wilcox test)
8.127043e−03
8.127043e−03
8.127043e−03
8.127043e−03
7.795827e−03
7.154777e−03
6.150611e−03
6.150611e−03
6.150611e−03
4.998125e−03
4.998125e−03
4.998125e−03
4.998125e−03
4.998125e−03
4.336913e−03
4.329004e−03
4.329004e−03
4.329004e−03
4.329004e−03
4.329004e−03
4.329004e−03
4.329004e−03
4.329004e−03
4.329004e−03
4.329004e−03
2.164502e−03
2.164502e−03
2.164502e−03
2.164502e−03
2.164502e−03
